# Effect of knee joint weight change on knee function recovery and gait after total knee arthroplasty

**DOI:** 10.1186/s12891-022-05647-5

**Published:** 2022-07-22

**Authors:** Zhengya Zhu, Tao Tang, Sheng Pan, Ziqian Sun, Chaoran Huang, Ruxin Ruan, Zhongyuan He, Shaoyu Liu, Xin Zheng, Kaijin Guo

**Affiliations:** 1 Department of Orthopaedics, Seventh Affiliated Hospital of Sun Yat-Sen University, 628 Zhenyuan Road, Shenzhen, 518107 People’s Republic of China; 2 Department of Orthopaedics, Affiliated Hospital of Xuzhou Medical University, 99 Huaihai West Road, Xuzhou, 221006 People’s Republic of China; 3 Department of Oncology, Xuzhou Cancer Hospital, Huancheng Road, Xuzhou, 221006 People’s Republic of China; 4 Department of Orthopaedics, Zhujiang Hospital of Southern Medical University, 253 Gongye Avenue, Guangzhou, 510282 People’s Republic of China

**Keywords:** Total knee arthroplasty, prosthesis, weight, Gait, HSS Score

## Abstract

**Background:**

Knee osteoarthritis (KOA) is a common disease based on degenerative pathological changes. Total knee arthroplasty (TKA) is an effective treatment for end-stage of KOA. However, only volume adaptation can be achieved with current knee prostheses, and it is difficult to achieve weight adaptation. This study focused on the weight difference of knee joints and initially explored the impact of this change on knee joint functional recovery and gait changes in patients after surgery.

**Methods:**

From October 2015 to June 2019, patients who underwent primary unilateral TKA were enrolled in this prospective cohort study with the same brand of knee prostheses. General data were collected from patients who met the criteria. The resected bone and soft tissues were collected and weighed precisely during TKA, and multivariate regression analysis was used to determine the factors affecting the weight of the removed knee tissues. We compared the weight of excised tissues and the total weight of the knee prosthesis, and the weight difference was defined as the increased weight of the knee joint (IWKJ). All patients were evaluated by HSS score, gait analysis, and affected side knee X-ray at two weeks, three months, and the last follow-up after the operation. To further determine the influence of IWKJ on postoperative functional recovery, the relationship between IWKJ, HSS score, and gait analysis was analyzed by univariate regression.

**Results:**

In total, 210 patients were eventually included in observation. All patients underwent postoperative follow-up for no less than two years. Multiple regression analysis showed that the course of the disease, body weight, and kellgren-Larencen stage(K-L stage)of the affected knee joint were independent factors affecting the weight of the removed knee tissues and were positively correlated with it. Univariate analysis showed that IWKJ was negatively correlated with HSS score at two weeks and three months after the operation. In addition, the values of spatiotemporal parameters and knee rotation ROM were negatively correlated with IWKJ two weeks after surgery, while outside food load response was positively correlated with IWKJ. Cadence, knee rotation ROM, and Ankle rotation ROM were negatively correlated with IWKJ, while outside food was positively correlated with IWKJ three months after surgery. At the last follow-up, only the hip rotation ROM was positively correlated with IWKJ.

**Conclusions:**

All Patients underwent TKA had varying degrees of increased knee weight. The increased weight was 298.98 ± 63.77 g. Patients' body weight, K-L staging, and disease duration are important factors that cause differences in resected knee tissue. Three months after the operation, the changes in knee joint weight had a negative correlation with the HSS score, which at the same time, it had varying degrees of linearity with gait parameters. However, the influence of weight diminished over time.

## Background

Knee osteoarthritis (KOA) is a common disease based on degenerative pathological changes. It affects about 10 million adults in the United States [1]. Total knee arthroplasty (TKA) is an effective treatment to relieve pain and improve physical function in patients with severe KOA [2, 3]. Currently, it is estimated that nearly 1.8–2.4‰ patients are treated with TKA in developed countries every year. Moreover, the demand in developed countries for TKA will increase by 26–69% before the middle of the twenty-first century according to previous statistics [4].

With the increasing number of KOA patients, the occurrence of knee joint replacement with medical implants is of great importance for an aging population. Previous studies have shown that there is no significant difference in the effect of the implant design on knee function. However, few reports have focused on the influence of the knee prosthesis weight on postoperative joint functional recovery. A prospective study showed that the weight of knee prosthesis was 3.94 times that of excised tissue. Further analysis showed that this change accounted for about 0.5% of total body weight. They suggest that the increased local weight of the knee may do harm to functional recovery after TKA [5]. Rossi pointed out that patients tend to overestimate the weight of implants and they may refuse to do functional exercises because of their inability to adapt to the increased knee weight in the short term [6]. The weight of implants directly corresponds to the material from which they are made of. However, in many cases, all available materials may not be light. Since most knee prostheses consist of metal materials whose density is obviously higher than that of bone and soft tissue, only volume adaptation, rather than weight adaptation, can be achieved by TKA [7]. The weight of the prosthesis should match the weight of the removed bone and soft tissue, which is in line with the goals of precision medicine. Otherwise, the placement of heavy knee implants may negatively affect postoperative recovery [8]. To the best of our knowledge, the effect of knee weight changes on knee function and gait after TKA has not been reported. Therefore, we conducted such a prospective study to determine whether the change in the knee joint weight affects the recovery of knee function.

## Methods

### General patient information

**Fig. 1:**
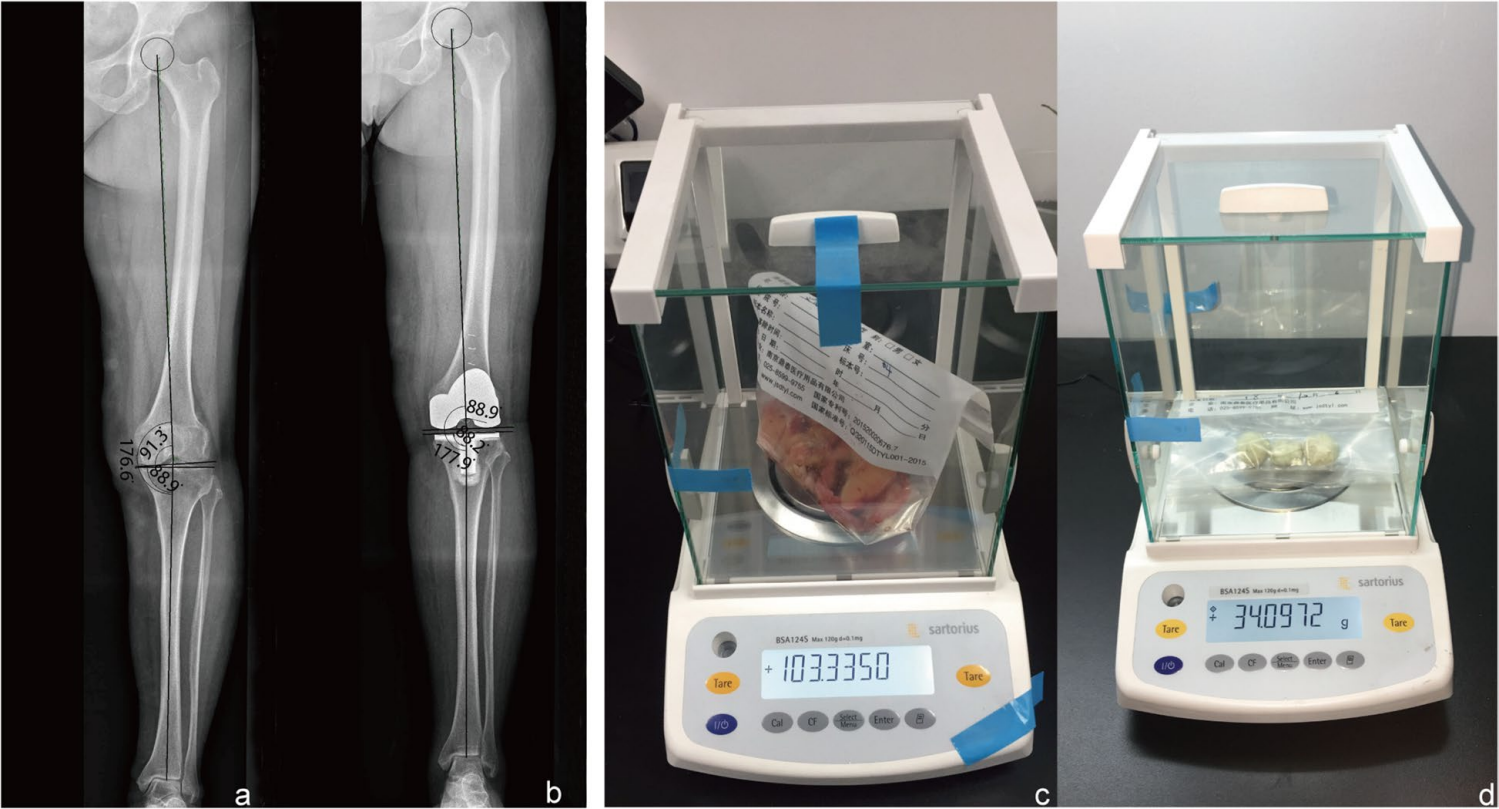
X-ray measurement of knee joint as well as weight measurement of excised knee tissue and unused bone cement. **a** HKA angle measurement before TKA; **b** HKA angle measurement after TKA; **c** weight of excised knee tissue; **d** weight of unused bone cement

Following approval from our institutional review board, patients who underwent unilateral primary TKA for KOA from October 2015 to June 2019 were eligible for enrolment in this prospective trial. Informed consent for participation was obtained from each patient before surgery. All patients involved in the study were required to register their age, sex, body mass index (BMI), course of the disease, visual analogue scale (VAS) score, and some other general information. All patients routinely underwent knee x-rays before surgery to determine the K-L staging of knee osteoarthritis, hip knee ankle (HKA) Angle, and the number of compartments affected [9, 10] (Fig. 1a-b).

### Inclusion and exclusion criteria

#### Inclusion criteria

Patients who met the following criteria were included: (i) initial TKA treatment; (ii) according to the condition, a prosthesis of the observed brand could be used; and (iii) no less than two years of follow-up.

### Exclusion criteria

Patients were excluded for the following reasons: (i) lack of follow-up data; (ii) patellar replacement was also performed; (iii) prior knee surgery; (iv) bilateral and simultaneous TKA; (v) use of additional prosthetic components, such as tibial extension rods; (vi) serious basal metabolic disease; and (vii) serious trauma resulting in prosthesis failure or secondary revision surgery.

### Operation method

All surgeries were performed by the same senior surgeon under general anaesthesia. Tourniquets.

were not routinely used unless patients were expected to suffer a prolonged operation or had a mild coagulation disorder (tourniquet pressure: 60 kPa). An anterior midline skin incision was made with a medial parapatellar approach. The hyperplastic synovium and part of the infrapatellar fat pad were removed. An intramedullary guide was used for the femur, and an extramedullary guide was used for the tibia. The bone surfaces were cleaned by high-pressure pulsatile lavage after osteotomy was completed. Then, the cement was injected under pressure into the cancellous bone to ensure better cement interdigitation, and a cemented knee joint prosthesis was installed. The patellar replacement was not performed in any patient.

### Weight of implanted and removed material

We defined bone and soft tissues removed during the operation as the removed material and the knee prosthesis and bone cement as the implanted material. The removed material and residual polymerized cement were collected using two specimen bags during the operation and then weighed using a precision electronic balance with an accuracy of 0.0001 g (Fig. 1c-d). The manufacturers provided the weight of the specimen bags, prostheses, and one full bottle of cement. The weight of the bone cement in the whole bottle minus the weight of the unused cement was calculated as the weight of the used bone cement. We defined the change of the knee joint weight as the total weight of the implanted material minus the total weight of the removed material.

In order to eliminate the influence of different implants on the postoperative efficacy of patients, all knees received cruciate-retaining (CR) implants (Sigma PFC®, DePuy, America). Otherwise, patients were excluded from this study. The femoral and tibial parts of the prostheses of this brand were made of cobalt-chromium (Co-Cr) alloy, and the spacer was made of highly cross-linked polyethylene.

### Multiple regression analysis of the influence on the weight of host removals during TKA surgery

Because the implant's weight is associated with shape, size, and material, it is hard for doctors to choose different weight implants. Therefore, we focused on the factors influencing the weight of removed bone and tissue during TKA surgery. In order to explore possible influencing factors, multiple regression analysis was performed with the weight of the removed bone and tissue as the dependent variable, and age, sex (male = 1, female = 2), BMI, K-L degree, preoperative HKA angle, and other factors as independent variables.

### Postoperative treatment

All patients adopted the same nutritional intervention, antibiotic treatment, thrombosis prophylactic, perioperative pain management, and nursing and rehabilitation treatment programs. Immediately after anaesthesia, all patients began to perform isometric contractions of the quadriceps femoris and pumping ankle movements. If the drainage volume was less than 50 ml 24 ~ 48 h after the operation, the drainage tube was removed, and then the dressing was changed every three days. The incision was monitored for signs of infection during dressing changes. All patients were instructed to start walking on the ground with the help of walking aids 48 h after the operation. In addition, all patients began to use a CPM machine to assist with knee functional exercise on the third day after surgery, which lasted for 30 min every day and was converted to independent exercise after one week.

### Observation of the curative effect at each time point postoperatively

All patients were evaluated by the Hospital for Special Surgery (HSS) score at two weeks, three months, and the last follow-up postoperatively to evaluate the knee joint recovery after TKA. HSS score is a reliable indicator for evaluating functional recovery after knee joint surgery. It can be used to describe the prognosis of patients in terms of pain, knee movement, and stability. HSS score was calculated by two surgeons who had more than three years of experience in orthopaedic surgery, and the final score was taken as the average of the two. If the results were quite different (differing by more than five points), a senior doctor was responsible for re-evaluation.

**Fig. 2:**
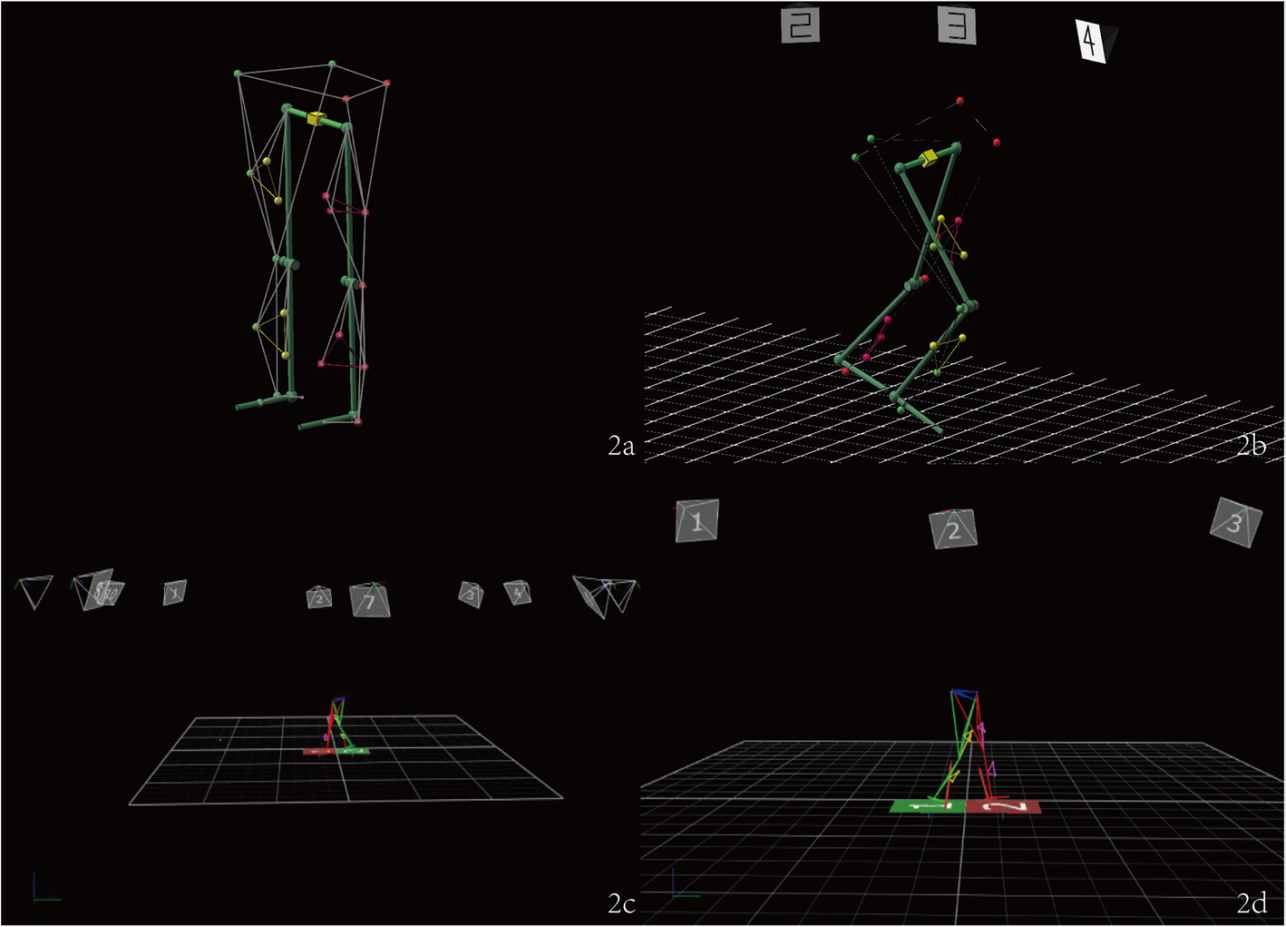
Computer simulation of 3D gait model. **a** Infrared reflective balls were attached to the lower limbs; **b** A grid plate could be used to calculate the walking distance. **c** Ten MX infrared cameras recorded the walking path. **d** Pressure plates were used to collect information on the patient's plantar reaction forces

In addition, gait analysis data of patients at different postoperative periods were collected to evaluate their walking trajectory and gait changes. At each time point after the operation, patients underwent a gait analysis by an optical motion capture system (Vicon MX, England), which is mainly composed of infrared reflective balls, 10 MX infrared cameras, and information conversion controllers. When the patient moves, the movement tracks of the infrared reflective balls on the patient's body are captured and recorded by the MX cameras (Fig. 2). The motion signals are analyzed by the information converters, and the results are obtained and quantized. According to the clinical trial design, three major outcomes of interest were collected: spatiotemporal parameters, kinematic parameters, and mechanical parameters. There were six observation indexes among the spatiotemporal parameters: walking speed (cm/s), cadence (stride frequency, step/min), step length (cm), single-foot support phase (%), stance phase (%), and swing duration (%). The kinematic parameters included three indexes: hip rotational ROM, knee rotational ROM, and ankle rotational ROM. The plantar reaction force parameter mainly describes the plantar reaction force and includes the reaction on the front side, rear side, inner side, outer side, and the vertical reaction force. The ratio of the maximum reaction force produced by each area to the reaction force on the sole in the static state is the peak reaction force in all directions. To reduce errors, all spatiotemporal parameters were the average values obtained when patients walked for at least three minutes.

### Univariate regression analysis between IWKJ and HSS score and gait analysis

HSS score and parameters of gait analysis were collected at two weeks, three months, and at the last follow-up after the operation. Pearson's correlation analyses were used to analyze the correlation between the weight of knee joint changes and HSS score and gait analysis.

## Sample size calculation

Correlations between the weight of knee joint changes and HSS score and indicators in gait analysis were assessed respectively using Pearson's correlation analyses. We assumed the correlation coefficient was ρ = 0.5, based on α = 0.05, β = 0.1 and a two-tailed test, according to the formula: *n* = 4 × {(Z_α/2_ + Z_β_) / ln [(1 + ρ) ∕ (1 − ρ)]}^2^ + 3 [11], and the total sample size should be no less than 165.

### Statistical analysis

Statistical analyses were performed using SPSS version 20 (SPSS, Chicago, USA). Quantitative data are presented as the mean and standard deviation (SD). Pearson's correlation analyses were used to analyze the correlation between the weight of knee joint changes and HSS score and gait analysis. Multivariate Linear regression analysis was used to describe the influencing factors on the weight of removed bone and tissue during TKA surgery. Statistical significance was set at *p* < 0.05.

## Results

### Preoperative demographic data of patients

**Fig. 3:**
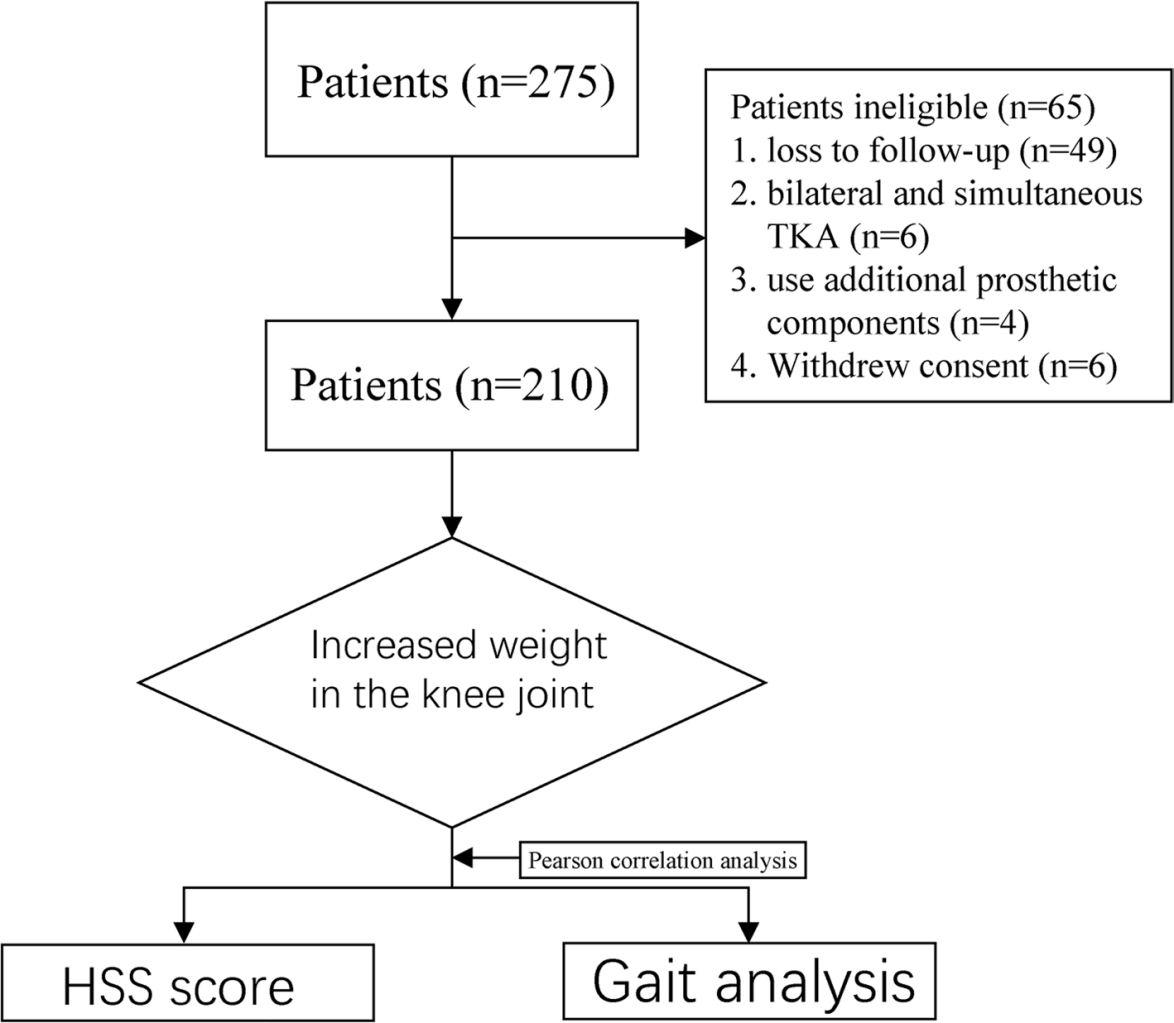
Flow diagram of enrolment. A total of 275 patients met the inclusion criteria of this study; 65 patients were excluded for various reasons, and 210 patients were eventually included in the analysis

A total of 275 patients met the inclusion criteria of this study; 65 patients were excluded for various reasons, and 210 patients were eventually included in the analysis (Fig. 3). All patients were followed up for at least two years. There were 56 males and 154 females, with an average age of 67.16 ± 7.31 years and an average BMI of 25.84 ± 3.75 kg/m^2^. According to the preoperative knee X-ray, the K-L stages of all patients' knees were III-IV grades. We measured the HKA angle of the patients to determine their deformity condition. 189 cases were of varus deformity and 18 cases were of valgus deformity (Table 1).

**Table 1:** Preoperative data of patients

Parameter	n	Average value	Range
Age	210	67.30 ± 7.40 (years)	49—85 (years)
Sex			
Male	56		
Female	154		
BMI	210	25.84 ± 3.75 (kg/m^2^)	19.31—36.79 (kg/m^2^)
Kellgren-Lawrence grade			
Grade III	47		
Grade IV	163		
Joint deformity			
Normal range/ HKA angle	3	177.71 ± 0.49°	177.29—178.25°
Varus/ HKA angle	189	168.74 ± 3.88°	162.14—176.83°
Valgus/ HKA angle	18	190.47 ± 4.54°	183.10—199.88°
VAS score	210	5.28 ± 2.44	1—9
HSS score	210	43.50 ± 12.04	10—62
Numbers of affected compartments			
Unilateral compartment	62		
Bilateral compartment	148		
Course of the disease	210	7.12 ± 4.90 (years)	3—20 (years)

### Changes in knee joint weight after TKA

**Fig. 4:**
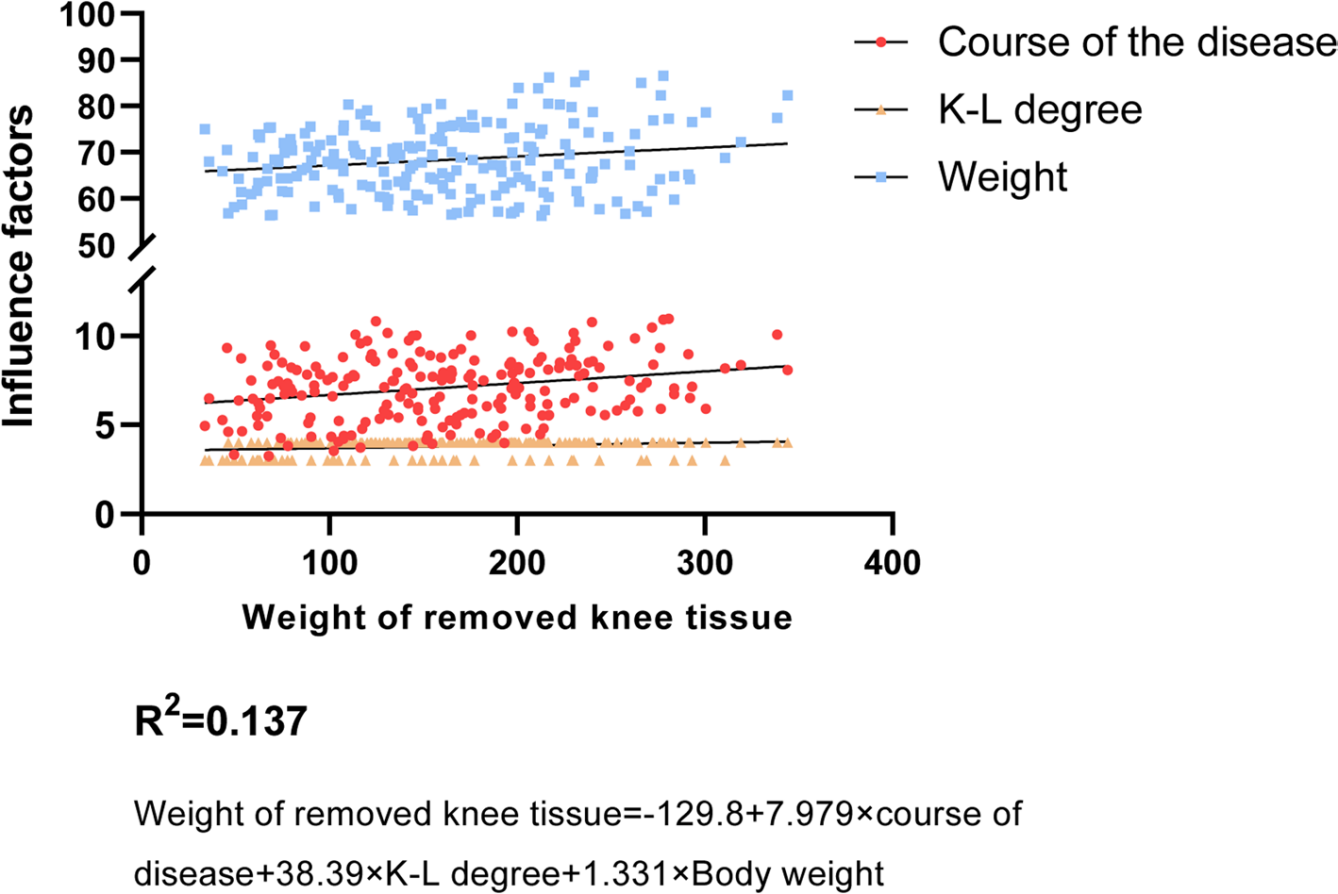
Multiple linear regression analysis of the weight of removed knee tissue

All patients enrolled in the study received the same brand of prostheses. The average total weight of prosthesis components was 453.71 ± 66.08 g (366.95 g—578.59 g), and the weight of implanted bone cement was 6.83 ± 2.70 g (2.06 g—11.98 g), and the weight of removed knee tissue was 162.70 ± 69.01 g (33.7 g—344.01 g). The increased weight of the knee joint was 298.98 ± 63.77 g (150.50 g—447.35 g). The results of multiple regression analysis showed that the k-L grade of the knee joint, the course of disease, and body weight were independent factors that affected the weight of the removed knee tissue (Fig. 4). Weight of removed knee tissue = -129.8 + 7.979 × course of disease + 38.39 × K-L degree + 1.331 × body weight.

### Univariate regression analysis between IWKJ and HSS score after TKA

**Fig. 5:**
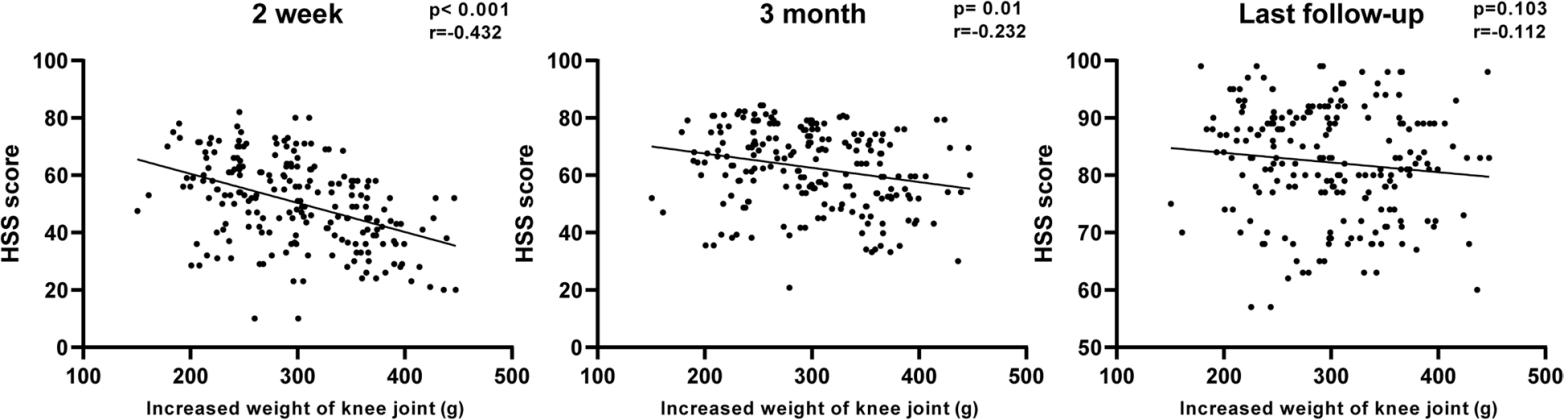
HSS score at each time point after TKA. In the early postoperative period, patients with lighter prostheses will get higher HSS scores. In the final investigation, there was no linear regression between the HSS score and IWKJ

The HSS score was evaluated in all enrolled patients at two weeks, three months, and the final follow-up after TKA. Two weeks after surgery, the patients' HSS scores were 50.47 ± 14.95 and the increased knee weight was negatively correlated with the HSS score (*p* < 0.001, *r* = -0.432). Three months after surgery, the patients' HSS scores were 62.66 ± 13.68 and the increased knee weight was also negatively correlated with the HSS score (*p* = 0.01, *r* = -0.232). At the last follow-up, the patients' HSS scores were 82.27 ± 19.16, and there was no significant correlation between increased knee weight and HSS score (*P* = 0.103) (Fig. 5).

### Univariate regression analysis between IWKJ and gait analysis data at different time points after TKA

#### Two weeks after TKA

**Fig. 6:**
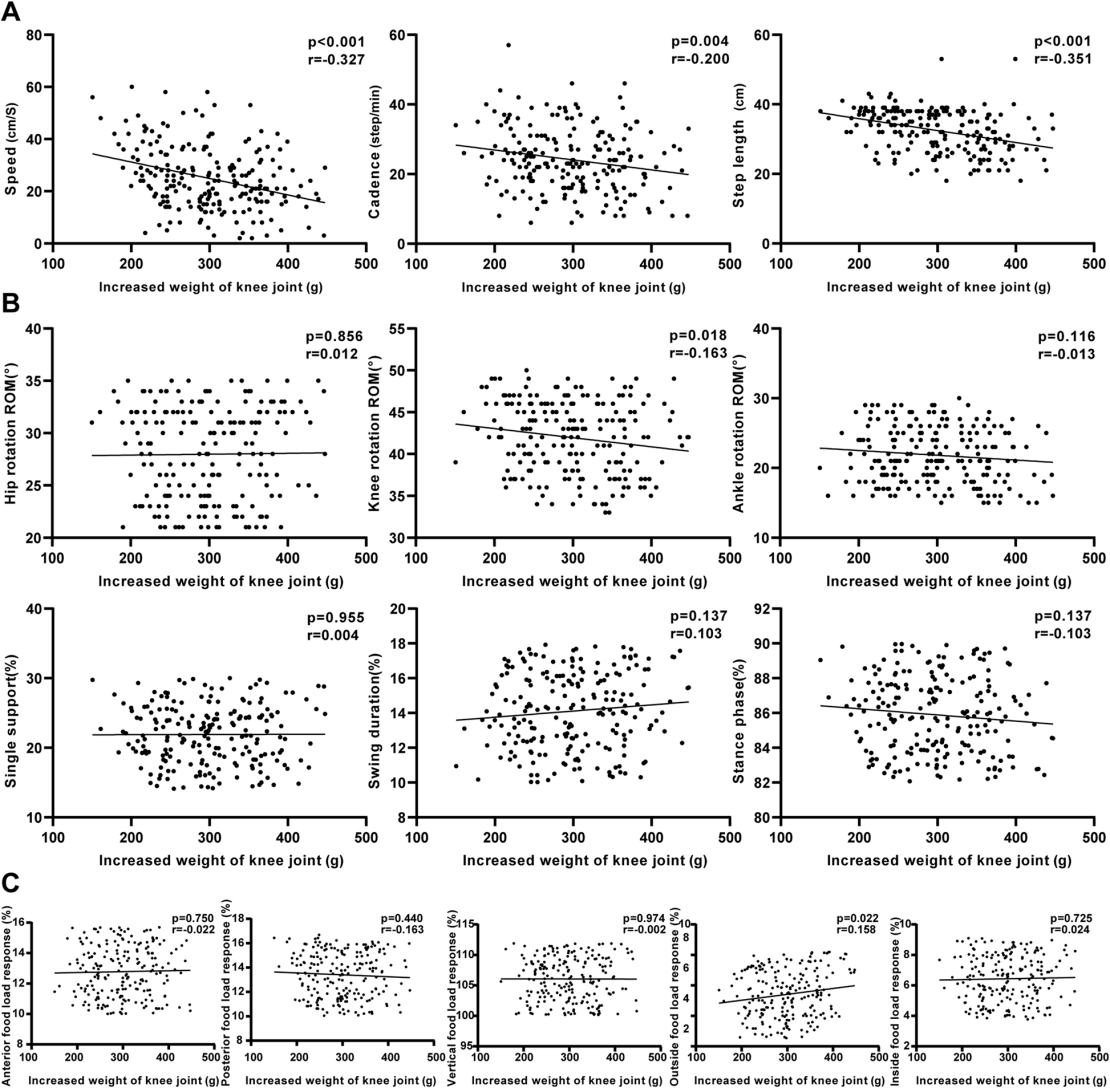
Two weeks after TKA, univariate regression analysis was performed between IWKJ and gait analysis. **A** linear relationship with spatiotemporal parameters; **B** linear relationship with kinematic parameters; **C** linear relationship with mechanical parameters

Two weeks after surgery, all the parameters of spatiotemporal parameters were negatively correlated with IWKJ (Fig. 6). The average stepping speed was 24.98 ± 12.28 cm/s (*p* < 0.001, *r* = 0.327); the average walk step per minute was 24.10 ± 9.08 (*p* = 0.004, *r* = 0.200), and each step length was 32.49 ± 6.19 cm (*p* < 0.001, *r* = 0.351). Among the kinematic parameters, the knee rotation ROM was negatively correlated with IWKJ (*p* = 0.018, *r* = -0.163). Among the mechanical parameters, outside foot load response was positively correlated with IWKJ (*p* = 0.022, *r* = 0.158). Other gait analysis parameters had no univariate linear relationship with IWKJ (*p* > 0.05).

#### Three months after TKA

**Fig. 7:**
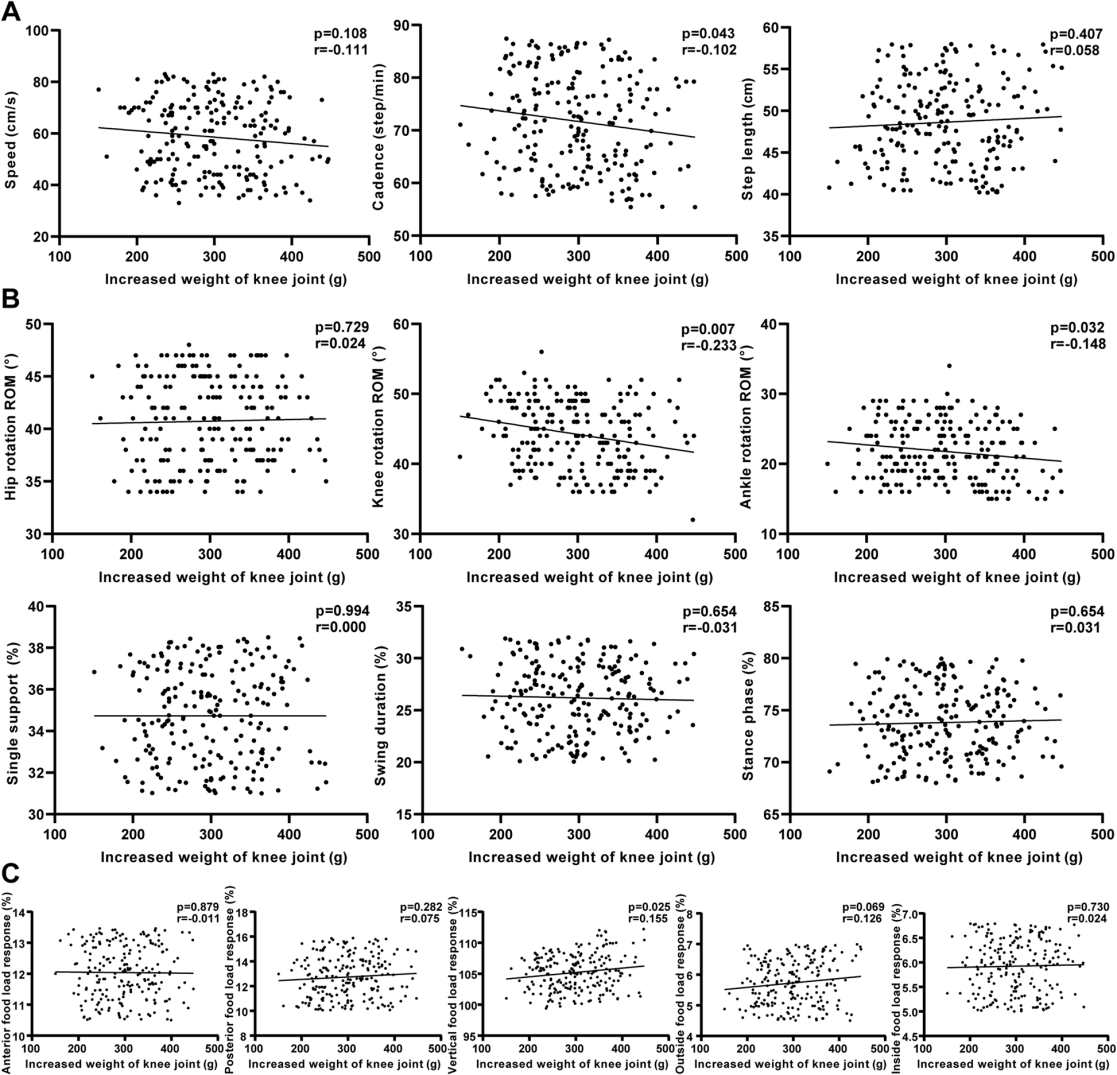
Three months after TKA, univariate regression analysis was performed between IWKJ and gait analysis; **A** linear relationship with spatiotemporal parameters; **B** linear relationship with kinematic parameters; **C** linear relationship with mechanical parameters

Three months postoperatively, some indexes of gait analysis were influenced by IWKJ (Fig. 7). Patients' walking cadence was 71.70 ± 9.23 steps per minute, which was negatively correlated with IWKJ (*p* = 0.043, *r* = -0.102). Among the parameters of kinematic, both the knee rotation ROM and ankle rotation ROM were negatively correlated with IWKJ. Besides, there was a positive correlation between vertical foot load response and IWKJ (*p* = 0.025, *r* = 0.155). Other gait analysis data had no univariate linear relationship with IWKJ (*p* > 0.05).

#### At the last follow-up

**Fig. 8:**
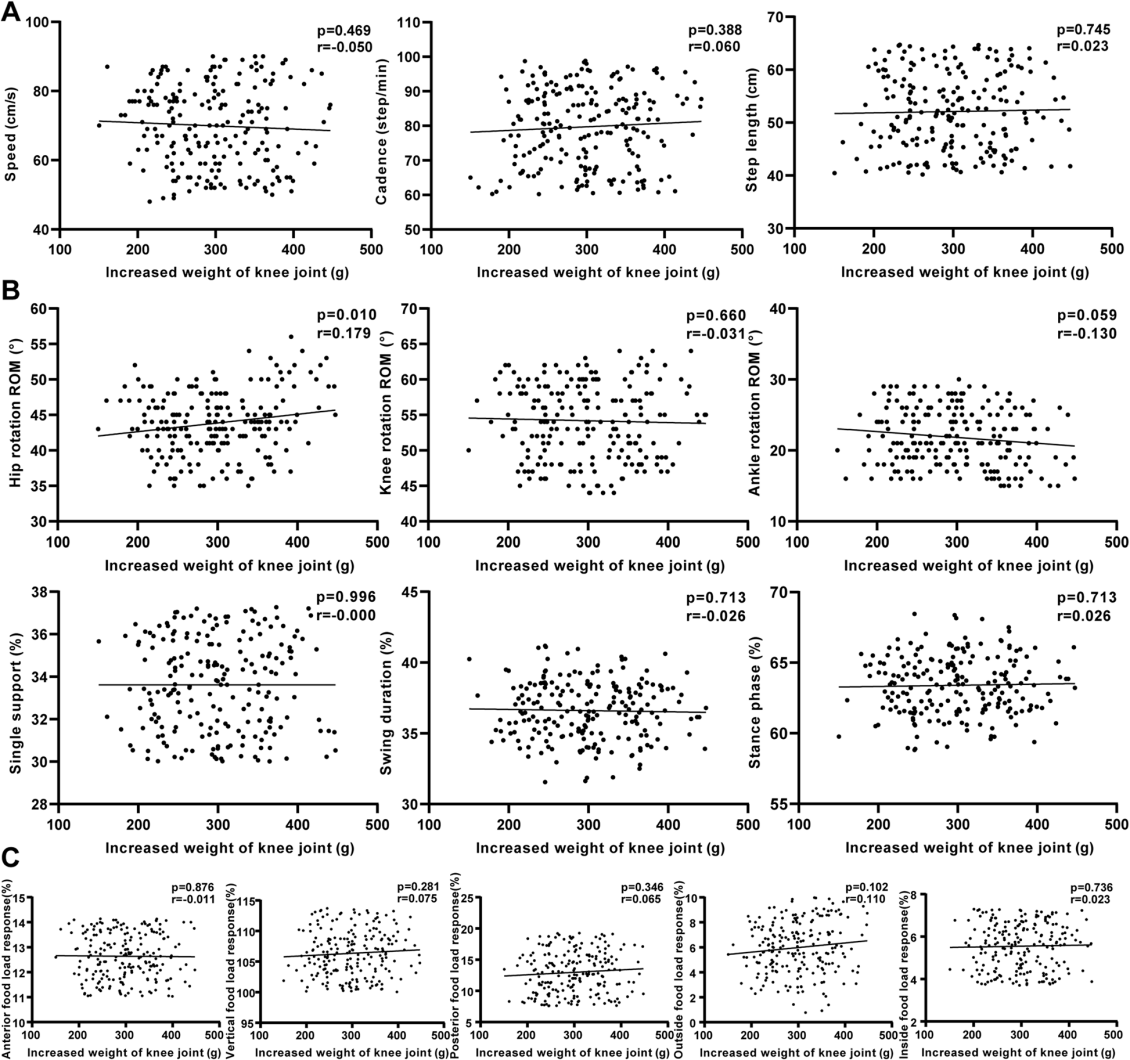
Last follow-up after TKA, univariate regression analysis was performed between IWKJ and gait analysis. **A** linear relationship with spatiotemporal parameters; **B** linear relationship with kinematic parameters; **C** linear relationship with mechanical parameters

We observed an interesting phenomenon that results at the last follow-up were completely different from those at two weeks and three months (Fig. 8). There was no clear linear regression relationship between each parameter of spatiotemporal parameters and mechanical parameters and IWKJ. In addition, there was no single factor correlation between the knee rotation ROM and the ankle rotation ROM and weight increase, while there was a positive correlation between the hip rotation ROM and IWKJ (*p* = 0.010, *r* = 0.179).

## Discussion

### TKA and weight of knee prosthesis

With the increasing demand for TKA, a full understanding of all parameters related to this surgery is essential [12]. There have been few reports on variation in the weight of the knee joint, an important parameter after TKA. Gibon et al. confirmed that the density of human bone and soft tissues is far less than those of metal prostheses. Therefore, it is inevitable that patients will get an additional weight gain in their knees when they receive implants of similar size instead of their natural knees [7]. The previous study has shown that the weight of knee prosthesis was 3.94 times that of excised tissue, and the increased weight of the knee joint accounted for 0.5% of the total body weight [5]. Although this weight is not important for the whole body, a sharp increase in local knee weight may have significant side effects on the recovery of knee function. In our study, the results showed that the weight of the knee joint increased by 150.08 to 447.35 g, which is basically consistent with the results of previous studies[7, 8]. Our study suggests that patients' body weight, K-L staging, and disease duration are important factors that cause differences in resected knee tissue. KOA condition is often positively correlated with the course of disease, and the higher K-L grade. The narrower the joint space and the more severe subchondral sclerosis in imaging. Therefore, the above two factors can directly reflect the osteophyte hyperplasia of the affected knee joint. In addition, body weight is also closely related to the severity of KOA. Several studies have pointed out that excessive body weight will cause changes in the mechanical load of the knee joint, and intermediate products of fat cell metabolism will destroy the cartilage of the knee joint [13]. Despite these facts, few clinicians pay attention to the problems caused by the increased weight of the knee joint, such as whether it will increase the risk of surgical complications or affect knee joint function. The main purpose of this prospective clinical study is to investigate the effect of knee weight changes on knee function and gait after TKA.

### Knee joint weight changes and the HSS score

The HSS score is often used to evaluate KOA severity or knee joint recovery after TKA[14]. It can be used to comprehensively describe a patient's knee joint function from many aspects. Our research shows that postoperative knee function and prosthesis weight were significantly negative correlation in the first three months, and after receiving a long time (more than two years) of functional exercise, the knee joint is gradually less affected by this change. The practical clinical problem is that the first three month is always the golden time for patients to exercise and recover. The poor functional recovery during this period will not only make patients under a certain psychological burden but also have a certain impact on the formation of postoperative joint stiffness and other complications later. Poor functional recovery of the knee joint in the early stage after TKA may be related to the decreased muscle strength around the knee after TKA. Ericsson et al. pointed out that with the progression of the KOA, the quadriceps muscle strength of patients gradually decreased [15]. In addition, a previous study has shown that the strength of the quadriceps muscle and hamstring muscle on the affected side knee joint was significantly lower than that on the healthy side after TKA [16]. At our last follow-up, almost all patients had significantly higher scores than that at two weeks after TKA. We believe that the score improvement is related to the specific content of the grading system. Two weeks after the operation, the patients were in poor physical condition, and most of them needed a walking aid. It was difficult for patients to walk for a long distance, or, up or downstairs, so the functional score at this time would be very low.

### Knee joint weight changes and gait analysis

Previous studies have shown a significant difference in gait on the affected side compared to the healthy side in patients after TKA [17]. We wanted to know whether this difference was related to the change in the knee joint weight. In addition, the spatiotemporal parameters of gait analysis can compensate for the deficiency of the HSS score. Meikle's research showed that there was no significant difference in the results of gait analysis in amputees wearing prostheses of different weights, and interestingly, patients were more inclined to choose heavier prostheses [18]. However, our research revealed contrary results: in the early stage after TKA, patients with heavier prostheses showed a slower pace and shorter stride. The main difference between our results and those reported by Ben is that their research objects were patients who had undergone amputation long before the study. The lower limb prosthesis was worn much like an article of clothing, and there was almost no difference in muscle strength between the affected limb and the healthy limb. Our research objects were patients who had recently undergone surgical treatment; at this time, the strength of the quadriceps femoris muscles and hamstring muscles of the affected side was significantly lower than that of the healthy side [19]. The difference in postoperative muscle strength may be an important factor affecting the gait changes of patients.

At the last follow-up, we observed an interesting phenomenon that there was no linear regression relationship between either spatiotemporal parameters or mechanical parameters and IWKJ, while hip joints gained a larger range of motion. We think it might have something to do with the muscles that control hip movement, which become stronger as patients tolerate heavier implants. This finding is consistent with Kim's [20]. In their latest research, a new prosthesis made of titanium (Ti) alloy was used in the clinic, and the weight of the prosthesis was similar to that of the removed material. Due to the limitations of material science and technology, we are now unable to perform TKA with lighter prostheses such as Ti alloy. However, I believe that with more attention paid to the weight parameter of the prosthesis by clinicians and manufacturers, lightweight prostheses will be widely available soon.

### Limitations

This study has some limitations. First of all, due to the lack of literature for reference, we defined the bone and soft tissue removed during TKA as the removed material. Obviously, after TKA, it is difficult for the bone to regrow in a short time, but we cannot judge whether soft tissues will return to the preoperative state in a short time given that the post-operative inflammatory state may lead to soft tissue hyperplasia and fibrosis. So the regeneration may be significant. This parameter might worsen the feeling of having a heavy knee after the procedure or might lead to poor outcomes. Although magnetic resonance imaging (MRI) can reflect soft tissue hyperplasia to some extent, most patients did not agree to undergo an MRI examination, so we could not obtain these data. There's another thing I have to point out about tissue weighing, which is that we didn't measure the synovial fluid in our study. When we opened the knee joint, it was very difficult to accurately collect the synovial fluid under certain pressure. We tried to calculate the synovial fluid with an aspirator, but the synovial fluid can easily infiltrate into the tissue or get mixed with the blood. In addition, during the process of expanding the pulp cavity in TKA, a small amount of bone powder cannot be collected. However, we believe that this amount of bone powder is not significant compared to the amount of bone collected. Second, due to the limitation of examination costs, we failed to collect the patients' gait analysis data before surgery, which could have a certain reference value for evaluating the patients' postoperative gait changes. In the following clinical observation, we will focus on this part. Finally, our longest follow-up time was less than six years, so long-term follow-up data to assess the long-term effects of changes in knee weight on knee function is lacking.

## Conclusion

All Patients undergoing TKA had varying degrees of increased knee weight. The increased weight was 298.98 ± 63.77 g. Patients' body weight, K-L staging, and disease duration are important factors that cause differences in resected knee tissue. Three months after the operation, the changes in knee joint weight had a negative correlation with the HSS score. Meanwhile, it had varying degrees of linearity with gait parameters. However, the influence of weight diminished over time.

## Data Availability

The datasets used and/or analysed during the current study are available from the corresponding author on reasonable request.

## Abbreviations

KOA: Knee osteoarthritis
TKA: Total knee arthroplasty
BMI: Body mass index
HSS: Hospital for special surgery
ROM: Range of motion
MRI: Magnetic resonance imaging
IWKJ: Increased weight of the knee joint
K-L grade: Kellgren and Lawrence grade
